# Perceived relative harm of heated tobacco products (IQOS), e-cigarettes, and cigarettes among adults in Canada: Findings from the ITC Project

**DOI:** 10.18332/tid/127233

**Published:** 2020-09-21

**Authors:** Edward Sutanto, Connor R. Miller, Danielle M. Smith, Richard J. O’Connor, Shannon Gravely, David Hammond, Andrew Hyland, Kenneth M. Cummings, Anne C. K. Quah, Geoffrey T. Fong, Thomas K. Agar, Maciej L. Goniewicz

**Affiliations:** 1 Department of Health Behavior, Roswell Park Comprehensive Cancer Center, Buffalo, United States; 2 Department of Psychology, University of Waterloo, Waterloo, Canada; 3 School of Public Health and Health Systems, University of Waterloo, Waterloo, Canada; 4 Department of Psychiatry and Behavioral Sciences, Medical University of South Carolina, Charleston, United States; 5 Ontario Institute for Cancer Research, Toronto, Canada

**Keywords:** heated tobacco product, electronic cigarette, cigarette, harm perception

## Abstract

**INTRODUCTION:**

Tobacco companies have introduced heated tobacco products (HTPs), such as IQOS, which may compete with e-cigarettes among smokers interested in switching to potentially reduced-risk products. Non-smokers may also start using IQOS if they believe this product is less harmful than other nicotine products. Smokers’ and non-smokers’ decisions may be driven by relative harm perceptions of emerging nicotine products. We aimed to examine relative harm perceptions between IQOS, e-cigarettes, and cigarettes, among nicotine product users and non-users.

**METHODS:**

We conducted a web survey with Canadian respondents (aged ≥20 years; n=268) in September–October 2018. Perceptions about relative harm between IQOS (available for sale since 2017 and subject to the same comprehensive marketing restrictions as cigarettes in Canada), e-cigarettes, and cigarettes, were assessed among non-users (n=79), exclusive smokers (n=78), exclusive e-cigarette users (n=32), and dual users (n=79). Multiple logistic regression explored the association between relative harm perceptions and nicotine-use status, adjusting for sociodemographic variables.

**RESULTS:**

Over half of respondents perceived IQOS as equally or more harmful than e-cigarettes (53.7%), while almost a quarter either reported IQOS as less harmful than e-cigarettes or were uncertain (22.7% and 23.5%, respectively). Two-thirds of respondents (65.7%) perceived e-cigarettes as less harmful than cigarettes, yet only half (48.1%) perceived IQOS as less harmful than cigarettes. Both exclusive and dual e-cigarette users, but not exclusive smokers, had higher odds of perceiving IQOS as more harmful than e-cigarettes and less harmful than cigarettes compared to non-users.

**CONCLUSIONS:**

Most nicotine users and non-users perceive differential health risk across IQOS, e-cigarettes, and cigarettes. Although e-cigarettes are generally viewed as less harmful than cigarettes, the perceived harm of IQOS was unclear.

## INTRODUCTION

Over the past decade, the novel nicotine product marketplace has expanded to accommodate growing interest in potentially reduced-risk alternatives to combustible-cigarettes (hereafter ‘cigarettes’). Independent companies have introduced electronic cigarettes (e-cigarettes) while tobacco companies re-introduced heated tobacco products (HTPs). The most notable brand of the latter being Philip Morris International’s IQOS. While many countries do not allow alternative nicotine products to be marketed as less harmful than cigarettes, there are often subtle messages about the potential risk reduction if smokers switched to e-cigarettes or HTPs. Lower harm perceptions toward certain nicotine products have been reported as a predictor of future use^1^. While many studies have examined harm perceptions of e-cigarettes relative to cigarettes^2,3^, only a handful have evaluated HTPs relative to cigarettes^4–6^, and none has assessed HTPs relative to e-cigarettes.

In Canada, where both e-cigarettes and HTPs were available for a substantial period, e-cigarettes became legally available for sale in May 2018^7^, while IQOS was first launched in April 2017^4^. Nearly 3% of Canadians aged ≥15 years reported past 30-day use of e-cigarettes in 2017^8^, which increased to 14.6% among Canadian adolescents in 2018^7^. While there have not been nationally-representative estimates for past 30-day use of HTPs in Canada, 6.4% of Canadian youth reported awareness of IQOS, and 33.0% expressed interest in trying the product in 2017^9^. Thus, we aimed to examine relative harm perceptions between IQOS, e-cigarettes, and cigarettes, among nicotine product users and non-users in Canada.

## METHODS

### Data source

We analyzed Canadian participant data from the International Tobacco Control Japan–Canada Heated Tobacco Products (ITC JCH) Project, a web-based survey conducted from September to October 2018. The ITC JCH Project used targeted recruitment via offline methodologies (a mix of panel usage, referrals, and consumer lists) in Canada. Japanese participants were not included in this study as the sale of e-cigarettes is banned in Japan, thus the survey did not ask about e-cigarette harm perceptions to Japanese participants. The survey screened potential participants for awareness to IQOS, e-cigarettes, and cigarettes; only those who were aware of all three products were eligible for the survey.

Based on nicotine-use status, adult (aged ≥20 years) residents of Canada (N=275) were recruited as: 1) Non-user (never use or has stopped use of any tobacco product for the past 12 months, n=79); 2) Exclusive smoker (daily use of cigarettes for the past 3 months, n=78); 3) Exclusive e-cigarette user (daily use of e-cigarettes for the past 3 months, n=32); or 4) Dual user of e-cigarettes and cigarettes (daily use of both cigarettes and e-cigarettes for past 3 months or daily use of e-cigarettes and weekly use of cigarettes for past 3 months, n=79). Although we recruited exclusive IQOS users (n=1) and dual cigarette-IQOS users (n=6), they were omitted from this study as there were not enough participants from either user group. Ethics approval was obtained from the Office of Research Ethics University of Waterloo and Roswell Park Comprehensive Cancer Center Institutional Review Board. Further details on the recruitment strategy are provided in the ITC JCH Survey Technical Report (https://itcproject.s3.amazonaws.com/uploads/documents/ITC_JCH_Technical_Report_FINAL-May8.pdf).

### Measures

We assessed participants’ perceived harm of IQOS compared to e-cigarettes, IQOS compared to cigarettes, and e-cigarettes compared to cigarettes using the following questions: ‘Compared to using e-cigarettes, how harmful do you think using IQOS is?’; ‘Compared to smoking ordinary cigarettes, how harmful do you think using IQOS is?’; and ‘Compared to smoking ordinary cigarettes, how harmful do you think using e-cigarettes is?’. In addition to ‘refused’ or ‘do not know’, five-point scales were provided (from ‘much more harmful’ to ‘much less harmful’) as options. For statistical analysis, responses were dichotomized as less harmful (‘much less harmful’ and ‘somewhat less harmful’) versus otherwise.

### Sociodemographics

Outcomes of interest were examined according to age and sex. Highest level of educational attainment was classified as: low (‘grade school/some high school’ and ‘completed high school’), moderate (‘technical/trade school or community college’ and ‘some university, no degree’), and high (‘completed university degree’ and ‘postgraduate degree’). Annual household income was classified as: low (<CA$29999), moderate (CA$30000–59999), high (≥CA$60000), and not reported (refused/do not know).

### Statistical analysis

Estimates for relative harm perceptions were presented as percentages. Multiple logistic regression explored associations between relative harm perceptions, nicotine-use status and sociodemographic variables. All statistical analyses were performed using Stata SE 15.1 (StataCorp). A p<0.05 was considered statistically significant.

## RESULTS

### Sample characteristics

Approximately half of respondents were male (50.7%). A greater proportion of respondents were aged 40–59 years (39.9%) and reported a moderate level of education (45.1%). Most respondents fell within the high-income category (≥ CA$60000) (Supplementary Table S1).

### Relative harm perceptions

**Figure 1:**
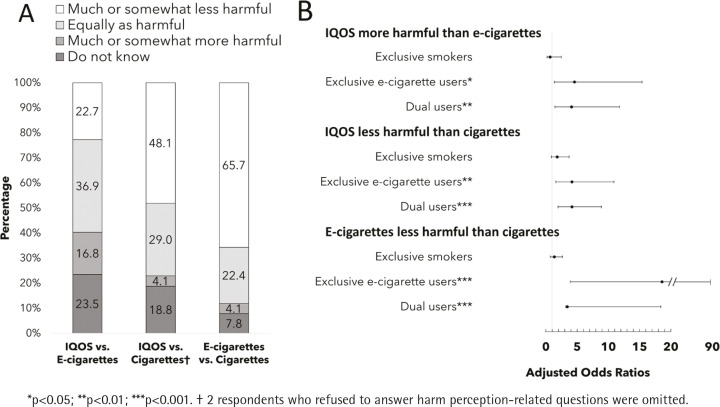
A. Overall relative harm perceptions of IQOS, e-cigarettes, and cigarettes (n=268). B. Adjusted regression analysis of relative harm perceptions by nicotine use status compared to non-users (reference group).

Figure 1A displays overall relative harm perceptions of IQOS, e-cigarettes, and cigarettes. More than one-third of respondents perceived IQOS to be equally as harmful as e-cigarettes (36.9%), almost a quarter either reported IQOS as less harmful or were uncertain(22.7% and 23.5%, respectively), while the rest perceived IQOS to be more harmful than e-cigarettes (16.8%). When respondents were asked to compare independently relative harms of IQOS and e-cigarettes to cigarettes, two in three respondents (65.7%) perceived e-cigarettes as less harmful than cigarettes, yet only half (48.1%) perceived IQOS as less harmful than cigarettes.

Figure 1B shows adjusted regression analysis of relative harm perceptions by nicotine-use status. Compared to non-users, exclusive e-cigarette users had higher odds of perceiving IQOS as more harmful then e-cigarettes (AOR=4.58; 95% CI: 1.36–15.38), IQOS as less harmful than cigarettes (AOR=4.17; 95% CI: 1.60–10.88), and e-cigarettes as less harmful than cigarettes (AOR=18.59; 95% CI: 3.90–88.54). Similarly, dual users had higher odds of perceiving IQOS as more harmful than e-cigarettes (AOR=4.11; 95% CI: 1.44–11.79), IQOS as less harmful than cigarettes (AOR=4.16; 95% CI: 1.95– 8.87), and e-cigarettes as less harmful than cigarettes (AOR=3.43; 95% CI: 3.17–18.41).

## DISCUSSION

To our knowledge, this is the first study to compare relative harm perceptions between IQOS and e-cigarettes. When both products were compared to each other, most respondents perceived them to be equally harmful or were uncertain. When the risk of each product was independently contrasted with cigarettes, more respondents perceived e-cigarettes than IQOS as less harmful. While HTPs have been promoted globally as less harmful than cigarettes^10^, no claims have been made by tobacco companies regarding relative harm of HTPs versus e-cigarettes. At the time of this study, relative harm claims for IQOS would have been prohibited in Canada with HTP being subject to the same comprehensive marketing restrictions as cigarettes^11^. Although major reviews suggested e-cigarettes could play a role in smoking harm reduction^12,13^, where HTPs fall within the harm continuum of cigarettes and e-cigarettes is less clear. Current evidence suggests that exposure to harmful and potentially harmful chemicals from HTPs may be lower than from cigarettes, though much of it is industry funded^13,14^. The United States Food and Drug Administration authorized the marketing of IQOS as a modified-risk tobacco product in July 2020^15^. This underscores the need to differentiate levels of harms between HTPs and e-cigarettes, along with subsequent public dissemination of that information.

A novel finding of our study is that harm perception of e-cigarettes relative to IQOS differed by nicotine-use status. Exclusive and dual e-cigarette users had higher odds of perceiving IQOS as more harmful than e-cigarettes compared to non-users. This suggests that, while current e-cigarette users perceived other alternative nicotine products as less harmful than cigarettes, they perceived the product that they are currently using as the least harmful alternative.

Consistent with previous research^3^, most respondents in our study perceived e-cigarettes as less harmful than cigarettes. Almost half of respondents in our study perceived IQOS to be less harmful than cigarettes, which is two-fold greater than an estimate reported in an earlier Canadian survey conducted eight months after IQOS had been released^4^. Our survey was conducted one and a half years after IQOS was released in Canada, thus IQOS marketing could have reached a wider audience. We found current e-cigarette users had higher odds of perceiving both IQOS and e-cigarettes as less harmful than cigarettes compared to non-users. Despite being the target consumer of IQOS^16^, exclusive smokers did not have higher odds of perceiving IQOS as less harmful than cigarettes compared to non-users. Prior research in Canada reported current smokers had lowerodds than non-smokers of perceiving IQOS as less harmful than cigarettes^4^.

### Limitations

Two limitations of our study are the relatively small sample and the targeted recruitment strategy, which may limit the generalizability of our findings. Due to the small number of exclusive and dual IQOS users, we were not able to investigate harm perceptions in these user groups. Additionally, due to relatively short period between the time of survey and availability of both e-cigarettes (legally) and IQOS, harm perceptions could have been influenced by lack of familiarity with these products, and may change over time owing to media exposure and personal experience. Specifically, the survey was conducted before the outbreak of e-cigarette or vaping product use-associated lung injury (EVALI), which may influence harm perceptions of e-cigarettes moving forward. Lastly, because this study asked about a specific brand of HTPs (IQOS), findings may be less generalizable to other HTP brands.

## CONCLUSIONS

Although e-cigarettes were generally viewed as less harmful than cigarettes, the perceived harm of IQOS was unclear. Given the importance of public understanding of the health risks of HTPs within the nicotine products landscape, future studies should seek to quantify the harm of HTPs relative to both cigarettes and e-cigarettes.

## CONFLICTS OF INTEREST

The authors have each completed and submitted an ICMJE form for disclosure of potential conflicts of interest. The authors declare that they have no competing interests, financial or otherwise, related to the current work. R.J. O’Connor reports personal fees and non-financial support from World Health Organization and from Food and Drug Administration. In addition, he reports grants, personal fees and non-financial support from the National Institutes of Health, outside the submitted work. D. Hammond reports that he has received research grants from public health and government agencies to study tobacco and nicotine use, and has provided expert testimony on behalf of governments and public health authorities in response to legal challenges from tobacco, cannabis and e-cigarette companies. K.M. Cummings reports having received payments as an expert witness on behalf of plaintiff’s in litigation against cigarette manufacturers. G.T. Fong reports that he has served as an expert witness on behalf of governments in litigation involving the tobacco industry. Finally, M.L. Goniewicz reports personal fees from Johnson & Johnson, outside the submitted work.
