# Supplementary material for: Perceived relative harm of heated tobacco products (IQOS), e-cigarettes, and cigarettes among adults in Canada: Findings from the ITC Project
Source: Tob Induc Dis. 2020 Sep 21;18:81. doi: 10.18332/tid/127233 (PMC7528265; doi:10.18332/tid/127233)
Supplement: Supplementary file 1 [file TID-18-81-s1.pdf]

Supplementary Material

Perceived relative harm of heated tobacco products (IQOS), e-cigarettes, and cigarettes among adults in Canada: Findings from the ITC Project

Supplementary Table S1. Sample characteristics and adjusted logistic regression analyses of relative harm perceptions for specified tobacco products (n=268).

	**Sample characteristics**	**IQOS vs. E-cigarettes** IQOS is more harmful than e-cigarettes		**IQOS vs. Cigarettes** IQOS is less harmful than cigarettes		**E-cigarettes vs. Cigarettes** E-cigarettes are less harmful than cigarettes	
	n (%)†	% ‡	AOR (95% CI)	% ‡	AOR (95% CI)	% ‡	AOR (95% CI)
**Tobacco Use Status**							
Non-user	79 (29.5)	7.6	Ref	29.1	Ref	44.3	Ref
Exclusive smoker	78 (29.1)	6.4	0.68 (0.19-2.46)	41.0	1.80 (0.88-3.72)	53.8	1.35 (0.68-2.68)
Exclusive e-cigarette user	32 (11.9)	31.2	**4.58 (1.36-15.38)***	65.6	**4.17 (1.60-10.88)****	93.7	**18.59 (3.90-88.54)*****
Dual user	79 (29.5)	30.4	**4.11 (1.44-11.79)****	65.8	**4.16 (1.95-8.87)*****	87.3	**3.43 (3.17-18.41)*****
**Sex**							
Male	136 (50.7)	22.1	Ref	53.7	Ref	69.8	Ref
Female	132 (49.3)	11.4	**0.46 (0.22-0.99)***	41.7	0.75 (0.43-1.28)	61.4	0.87 (0.48-1.58)
**Age**							
20-29 years	38 (14.2)	15.8	Ref	63.2	Ref	76.3	Ref
30-39 years	104 (38.8)	19.2	1.70 (0.58-4.92)	55.8	0.88 (0.40-1.98)	72.1	1.13 (0.43-3.00)
40-59 years	107 (39.9)	16.8	1.75 (0.59-5.22)	40.2	0.55 (0.24-1.24)	58.9	0.86 (0.33-2.28)
60+ years	19 (7.1)	5.3	0.87 (0.08-8.90)	15.8	**0.22 (0.05-0.94)***	47.4	0.74 (0.20-2.74)
**Education**							
Low	45 (16.8)	13.3	Ref	46.7	Ref	71.1	Ref
Moderate	121 (45.1)	21.5	1.82 (0.60-5.52)	46.3	1.02 (0.47-2.26)	67.8	0.74 (0.31-1.76)
High	102 (38.1)	12.7	1.43 (0.41-5.02)	50	1.59 (0.68-3.72)	60.8	0.87 (0.34-2.17)
**Income**							
Low	22 (8.2)	22.7	Ref	50	Ref	63.6	Ref
Moderate	86 (32.1)	20.9	0.71 (0.20-2.48)	51.2	0.91 (0.33-2.52)	76.7	2.17 (0.69-6.85)
High	155 (57.8)	13.5	0.40 (0.11-1.46)	46.4	0.76 (0.28-2.07)	60.6	1.10 (0.37-3.34)
Not reported	5 (1.9)	20	0.71 (0.05-9.53)	20	0.20 (0.02-2.49)	40	0.39 (0.04-3.96)

† represents column percentage; ‡ represents row percentage.

* $p < 0.05$; ** $p < 0.01$; *** $p < 0.001$

AOR=adjusted odds ratios; CI=confidence interval; Ref=reference category.

© 2020 Sutanto E. et al.
